# Indole-3-Carbinol and Its Derivatives as Neuroprotective Modulators

**DOI:** 10.3390/brainsci14070674

**Published:** 2024-07-02

**Authors:** Alka Ashok Singh, Dhananjay Yadav, Fazlurrahman Khan, Minseok Song

**Affiliations:** 1 Department of Life Sciences, Yeungnam University, Gyeongsan 38541, Republic of Korea; alkasingh10f@gmail.com (A.A.S.); dhanyadav16481@gmail.com (D.Y.); 2 Institute of Fisheries Science, Pukyong National University, Busan 48513, Republic of Korea; fkhan055@pknu.ac.kr; 3 International Graduate Program of Fisheries Science, Pukyong National University, Busan 48513, Republic of Korea

**Keywords:** neurodegenerative, oxidative stress, indole-3-carbinol, antidepressant drugs, neuroprotective, derivatives

## Abstract

Brain-derived neurotrophic factor (BDNF) and its downstream tropomyosin receptor kinase B (TrkB) signaling pathway play pivotal roles in the resilience and action of antidepressant drugs, making them prominent targets in psychiatric research. Oxidative stress (OS) contributes to various neurological disorders, including neurodegenerative diseases, stroke, and mental illnesses, and exacerbates the aging process. The nuclear factor erythroid 2-related factor 2 (Nrf2)-antioxidant responsive element (ARE) serves as the primary cellular defense mechanism against OS-induced brain damage. Thus, Nrf2 activation may confer endogenous neuroprotection against OS-related cellular damage; notably, the TrkB/phosphoinositide 3-kinase (PI3K)/protein kinase B (Akt) pathway, stimulated by BDNF-dependent TrkB signaling, activates Nrf2 and promotes its nuclear translocation. However, insufficient neurotrophin support often leads to the downregulation of the TrkB signaling pathway in brain diseases. Thus, targeting TrkB activation and the Nrf2-ARE system is a promising therapeutic strategy for treating neurodegenerative diseases. Phytochemicals, including indole-3-carbinol (I3C) and its metabolite, diindolylmethane (DIM), exhibit neuroprotective effects through BDNF’s mimetic activity; Akt phosphorylation is induced, and the antioxidant defense mechanism is activated by blocking the Nrf2-kelch-like ECH-associated protein 1 (Keap1) complex. This review emphasizes the therapeutic potential of I3C and its derivatives for concurrently activating neuronal defense mechanisms in the treatment of neurodegenerative diseases.

## 1. Introduction

In the brain, oxidative stress (OS) is an imbalance in the production of reactive oxygen species (ROS) and the cellular capacity to neutralize them using antioxidants [1]. This imbalance is influenced by factors, such as excitotoxicity, diminished cellular antioxidant defenses, susceptibility of lipid-rich membranes to oxidation, and elevated oxygen requirements [2,3]. Excessive exposure to ROS causes functional and structural modifications in cellular biomolecules found within cells, including proteins, DNA, and lipids, which may compromise neuronal viability and function [4,5]. These mechanisms include mitochondrial dysfunction [6], increased generation of ROS and reactive nitrogen species (RNS), inadequate antioxidant defenses, protein oligomerization, and cytokine production, leading to inflammatory responses, abnormalities in the blood–brain barrier (BBB), and impaired proteasome function [7,8]. These dysfunctions are linked to the pathogenesis of neurodegenerative diseases. But the specific mechanisms underlying the pathobiologies of neurodegenerative disorders (NDDs) remain unclear. A range of clinical disorders and human diseases, including neurodegenerative disorders, can be caused by biochemical changes in these macromolecular constituents. The hallmark of neurodegenerative disorders is the progressive loss of particularly vulnerable neuronal cells [9], frequently concomitant with cytoskeletal protein aggregates that accumulate inside neurons and/or glial cells. These clumps are in the form of inclusions and are a common characteristic of neurodegenerative diseases. OS is closely linked to aberrant aggregated protein deposition and disruption of the metal ion balance [10]. Mitochondrial oxidative phosphorylation is the primary source of ROS [11]. OS, either directly or indirectly, increases the risk of neuronal death owing to malfunctioning mitochondria, alterations in physiological neurotransmitter metabolism, inflammation, proteostasis, or dysregulation of the antioxidant system. Most neurological conditions, such as Alzheimer’s disease (AD), Parkinson’s disease (PD), Huntington’s disease (HD), and amyotrophic lateral sclerosis (ALS) are frequently associated with elevated levels of RNS and ROS, and impaired antioxidant defense mechanisms [12]. Aging is by far the biggest risk factor for neurodegenerative illnesses, such as AD, PD, and ALS. It has been suggested that the buildup of mitochondrial DNA (mtDNA) and the net generation of ROS accelerate aging [13,14,15,16]. Immune system changes throughout aging are complicated and pleiotropic, indicating restructured or modified control, rather than a simple immune deficit. The immune system’s T cell compartment, which defends the body from infections and malignancies, has the most drastic alterations with age, which is coherent with the elderly’s greater incidence and severity of cancer and infections [17]. Moreover, the lipid-rich brain is susceptible to OS due to its high sensitivity to lipid peroxidation and comparatively weaker antioxidant defense. The unpaired valence electrons of ROS show extreme reactivity and the ability to harm cellular macromolecules [18,19].

**Figure 1:**
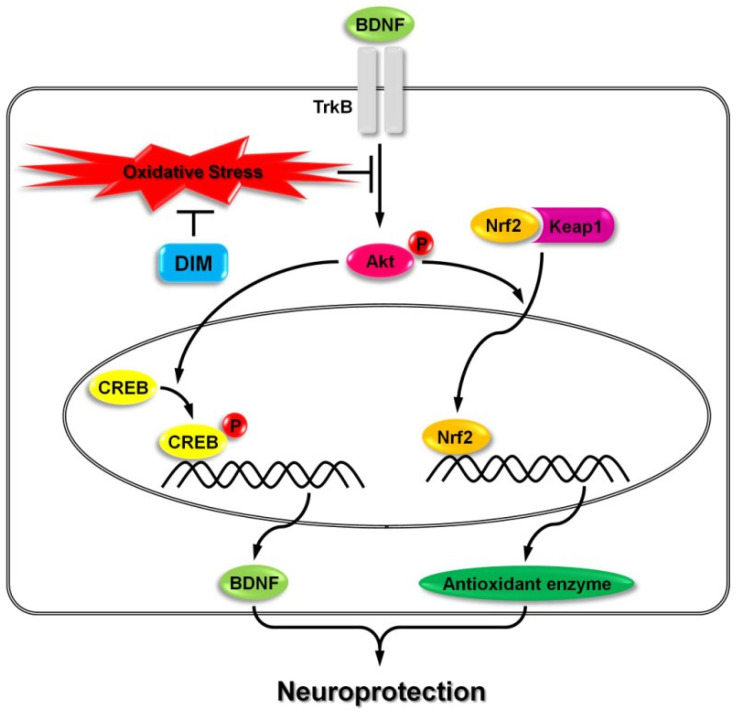
The role of DIM is to decrease oxidative stress and promote neurological function through BDNF and TrkB interaction. Reprinted from [21], Copyright © 2019 by the authors and Licensee MDPI, Basel, Switzerland.

DIM has previously been suggested as able to prevent oxidative stress-induced apoptosis in hippocampal neuronal cells, and its neuroprotective and antioxidant activities are closely related to the production of BDNF and antioxidant enzymes in these cells via stimulation of the TrkB/protein kinase B (Akt) pathways (Figure 1). This effect of DIM may aid the understanding of the neuroprotective function of indole-3-carbinol and N-palmitoyl serotonin-protected neuronal cells from oxidative stress-induced apoptosis by maintaining the BDNF autocrine loop, but it does not directly trigger TrkB phosphorylation [20].

## 2. Understanding Brain-Derived Neurotrophic Factor (BDNF) and Tropomyosin Receptor Kinase B (TrkB) Signaling in the Brain

Genetic predispositions, environmental factors, neuronal dysfunction, neurotransmitter imbalances, neuroinflammation, protein misfolding and aggregation, oxidative stress, mitochondrial dysfunction, impaired synaptic plasticity, and disruptions in neuronal networks are some of the factors that intricately interact to unravel brain disorders. Determining the fundamental causes of brain illnesses and creating successful treatment plans requires an understanding of the underlying pathways. To promote the survival of current neurons and their maturation and differentiation, BDNF influences specific neurons in the peripheral and central nervous systems that express TrkB [22,23]. BDNF–TrkB signaling stimulates cortical progenitor cell differentiation during embryogenesis, and subsequently stimulates the development of neurons from cortical progenitor cells into neurons (i.e., neurogenesis) [24]. Among the multiple neurotrophins, BDNF exhibits a high degree of gene function and structural conservation across vertebrates and is an important regulator of synaptic plasticity during brain development [25]. Astrocytes, microglia, and postsynaptic dendrites are the additional synapse components that can produce and secrete BDNF [26]. During the different stages of synaptic development, transcription, translation, and protein trafficking are facilitated by BDNF–TrkB signaling, which has also been linked to multiple types of synaptic plasticity [27]. By binding to TrkB, BDNF activates the following three canonical signaling pathways that support neuronal survival and synaptic plasticity: mitogen-activated protein kinase (MAPK), phospholipase C-γ (PLCγ), and phosphoinositide 3-kinases (PI3K)/protein kinase B (Akt) [28,29]. An earlier study reported the possible curative implications of BDNF and its effects on several neurological disorders [30]. Neuronal processes are mediated by the activation of the following three receptor tyrosine kinases belonging to the tropomyosin-related kinase family: TrkA (specific for nerve growth factor (NGF)), TrkB (for BDNF and neurotrophin-4 (NT-4)), and TrkC (for NT-3). Notably, the p75 neurotrophin receptor (p75NTR) was initially found to have a minimal affinity for each neurotrophin [31].

Neurogenesis, which is the process of creating functioning neurons from precursors, was once thought to only happen in the embryonic and perinatal phases of a mammal’s life [32]. The study of it has been considerably advanced within the past ten years in nearly all facets of adult neurogenesis in the mammalian central nervous system, driven by widespread interest and facilitated by methodological developments. Under normal circumstances, adult neurogenesis is spatially restricted to two distinct “neurogenic” brain regions: the subventricular zone (SVZ) of the lateral ventricles, where new neurons are generated and subsequently migrate through the rostral migratory stream (RMS) to the olfactory bulb to become interneurons, and the subgranular zone (SGZ) in the dentate gyrus of the hippocampus, where new dentate granule cells are generated [33]. The process of adult neurogenesis is dynamic and intricate, and it can be influenced by a range of physiological, pathological, and pharmacological inputs [34].

### 2.1. PI3K/Akt

PI3K and Akt are activated by various stimuli, including insulin, growth factors, cytokines, and cellular stress. This pathway serves as a crucial link between amyloid-β, neurofibrillary tangles, and brain atrophy [35]. Akt mediates several processes, including protein translation and cell viability. Mammalian target of rapamycin (mTOR), the essential regulator of protein synthesis, is regulated by a cascade activated by the TrkB-PI3K-Akt pathway [36]. Multiple diseases, including brain tumor metastasis and neurodegenerative disorders, are associated with the deregulation of the PI3K/Akt signaling pathway [37]. Activated Akt can phosphorylate and activate various substrates located in the nucleus and cytoplasm that are essential for many cellular processes, including migration, proliferation, differentiation, survival, glucose absorption and metabolism [38]. Furthermore, as evidenced by the functional deficits observed in several leukocyte subsets when PI3K and Akt are eliminated in experimental mouse models, Akt activation is crucial for triggering immunological responses [39,40]. Despite promoting aberrant microglial responses, the dysregulation of PI3K/Akt signaling also promotes neuronal damage and persistent neuroinflammation. When stimuli in their surroundings reach resting microglia, they are activated. This results in nitric oxide (NO) production, along with the coordinated expression of pro- and anti-inflammatory cytokines to neutralize the stimulus and aid recovery [41]. The PI3K/Akt signaling pathway is necessary for healthy animal development and growth. Furthermore, this pathway has been shown to be adequate for trophic factor-induced neuronal survival, and is crucial for the development of the CNS. By attaching to appropriate tyrosine kinase receptors, PI3K is activated by trophic factors, such as BDNF, NGF, and insulin-like growth factor (IGF1) [42,43]. In contrast, overexpression studies showed that Akt1 enhances the restoration of brain function and survival of human brain-derived neural stem cells (hNSCs) in a rat model of intracerebral hemorrhagic stroke, and confers neuroprotection on hNSCs impacted by OS in vitro [44]. Additionally, Akt1 improves the survival of neural cells in vivo and in vitro, and might shield the retina from light stress [45,46,47]. Collectively, these findings demonstrate the key role of Akt in facilitating the development and survival of neurons, both of which are essential for brain development.

### 2.2. MAPK

A family of serine–threonine kinases, known as MAPKs, is phosphorylated in response to certain mitogenic signals. Activation of MAPK induces the transfer of extracellular signals, derived from various receptors in the membrane, to intracellular targets, such as transcription factors, cytoskeletal proteins, and extracellular enzymes.

The tyrosine kinase receptor-mediated pathway sets off a phosphorylation sequence that activates adapter molecules. These, in turn, activate a guanine nucleotide exchange factor and small GTP-binding proteins, such as p21ras. This is observed in MAPK signaling, which is stimulated by growth factors, such as NGF [48]. The MAPK family includes P38 MAPK, c-Jun N-terminal kinase (JNK), and the extracellular signal-regulated kinase (ERK) family of signaling pathways that are impacted by amyloid-beta (Aβ) aggregation. These kinases control several biological functions, including differentiation, proliferation, and apoptosis [49]. Phospho-p38 MAPK level downregulation is associated with reduced neuroinflammation, which can counteract reactive gliosis and the excess production of cytokines that promote inflammation, such as interleukin (IL)-1β, IL-6, and tumor necrosis factor (TNF)-α. In human amyloid precursor protein/human presenilin 1 (APP/PS1) mice aged 11–12 months, a recent study using the p38α MAPK inhibitor MW150 showed that, while microglial cell counts were increased around the plaque, IL-1β and TNF-α levels were reduced [50]. An essential part of the innate immune system, the NOD-like receptor protein 3 (NLRP3) inflammasome mediates the activation of caspase-1 and the release of proinflammatory cytokines, IL-1β/IL-18, in response to microbial infection and cellular injury [51]; the activation of NLRP3 inflammasome results in the hyperactivation of p38/MAPK signaling, which leads an injured brain to continuously release various proinflammatory cytokines [52,53]. OS is a significant contraindication to AD commencement and progression [14,54]. It is a common stimulator of the JNK and p38 signaling pathways in AD, and is frequently induced by ROS, such as hydroxyl radicals, superoxide anions, and hydrogen peroxide [55]. Despite a range of processes, including the activation of neuronal death, activated MAPK signaling pathways are thought to contribute to the pathogenesis of AD [56]. The MAPK pathway promotes neuroprotection by initiating signaling cascades that control numerous physiological functions essential for neuronal survival. This route improves cell survival by inducing repair processes, regulating OS and inflammatory responses, and promoting the production of anti-apoptotic genes. Furthermore, MAPK signaling can promote synaptic plasticity, which is necessary to preserve the connection and functionality of neurons. Overall, protecting neurons from different insults and enhancing their survival under difficult circumstances is greatly aided by the activation of the MAPK pathway.

### 2.3. PLC-γ

PLCγ1 is a member of the PLC superfamily and is a cell growth factor [57]. PLC-γ1 is extensively expressed in the brain, and functions in neurotrophin-mediated neuronal cell activities. In line with PLC-γ1’s essential processes, it contributes to neural development and synaptic transmission [58]. Additionally, the PLC-γ pathway initiates once the TrkB receptor is activated. This pathway is believed to activate the inositol 1,4,5,-trisphosphate (IP3) receptor, which releases intracellular calcium reserves [59]. Since PLCγ1 can produce Ca2þ signals, it is crucial for the modulation of Ca2þ vacillation, the ablation of neurotransmitters, and modifications to synapse structure.

Since PLCγ1 expression patterns are closely linked to the onset of unprompted Ca2þ oscillation throughout neuronal maturation [60], under neurotrophin signaling PLCγ1 is crucial for controlling neurotransmission at the pre- and postsynaptic levels. Presynaptic PLCγ1 increases reverse transmission through the glutamate transporter and vesicle-mediated release to control the quantal exocytosis of glutamate [61]. Major pathways that are triggered by Trk receptors include PLCγ1, PI3K, Ras, and Rac, along with their downstream effectors [62]. In response to BDNF, TrkB’s intrinsic tyrosine kinase phosphorylates (activates) the phosphotyrosine residues on the protein, which attracts PLC-γ1 and induces downstream ERK2 and PI3K by binding to adaptor proteins like Shc and N-Shc [63]. The ras-dependent PI3K/Akt pathway that promotes survival is also triggered by Shc–Grb–2–Gab-1 scaffold proteins [64]. Because of its structural characteristics, growth factor receptor-bound protein 2 (GRB2) can participate in cellular activities. GRB2 is attracted to phosphorylated tyrosine residues on receptor tyrosine kinases (RTKs) through its SH2 domain during ligand activation. The Son of Sevenless (SOS) protein binds to GRB2 through its SH3 domain. This interaction activates Ras and initiates downstream signaling pathways that control cell division, proliferation, and survival [65]. The PLC-γ-specific array (γSA) is a unique region found in PLC-γ1 and PLC-γ2 in mammals. This region is bound by a split PH domain, two consecutive SH2 domains, and an adjacent SH3 domain. It is widely recognized that the SH2 and SH3 domains help PLC-γ interact with various molecules, supporting their physiological roles [58]. According to numerous studies, PLC-γ1 has been implicated in neurotrophin signaling and a number of neuronal processes, including synaptic plasticity, neurite outgrowth, and cell migration. PLC-γ1 is essential for regulating neurotransmission at pre- and postsynaptic levels in response to neurotrophin signals. It enhances glutamate release at the presynaptic sites by promoting reverse transmission through glutamate transporters and vesicle-mediated release. By mobilizing Ca^2+^ from IP3-sensitive stores and facilitating influx via transient receptor potential canonical (TRPC) channels, the release of PLC-γ1 boosts neurotransmitters, particularly glutamate, by enhancing vesicle fusion [61]. The development of neurons and neural networks is facilitated by the neuron-specific activation of PLCγ1 [66]. Abnormal expression and activation of PLC-γ1 occurs in brain disorders like depression, epilepsy, HD, and AD, indicating that it is involved in both neuronal processes and related brain disorders.

## 3. The Cellular Source of BDNF

Because the fate of secreted BDNF may influence variations in neuronal responses, it is necessary to understand the secretion and intracellular dynamics of BDNF proteins. ProBDNF and mature BDNF are secreted by neurons [67]. Live imaging of BDNF-green fluorescent protein (GFP) in neurons showed that 60% of BDNF is rapidly transferred anterogradely, which is consistent with earlier research suggesting that BDNF is sorted into dense-cored vesicles (DCV) and released during depolarization. These findings provide evidence for a paradigm in which synaptic plasticity can be modulated by targeting and releasing BDNF isoforms, most likely pro and mature BDNF [68]. Similar to BDNF, proBDNF is released by neurons following an action potential [68]. In addition, it plays an active biological role that conflicts with the prosurvival role of BDNF. Notably, proBDNF inhibits dendritic arborization, causes neuronal death, and adversely regulates synaptic transmission and plasticity [69].

BDNF was initially found in the brain, but is now known to be present in the blood as well, where it is effectively stored in platelets [70]. Because platelets have BDNF levels that can be 100–1000 times higher than those of neurons, they are the primary peripheral source of BDNF [71]. The proBDNF/BDNF ratio exhibits alterations within neurons or the cerebrospinal fluid in several neurocognitive conditions, including AD [72]. In a similar manner to neurons, BDNF is primarily stored in α-granules within platelets [73], and is released into the circulation upon platelet activation [70]. Platelets contain several neurotransmitters, such as glutamate, γ-aminobutyric acid (GABA), serotonin, adrenaline, dopamine, and histamine, which are vital for intercellular communication among brain cells. This implies that platelets may function as crucial messengers between the brain and peripheral organs by sending and receiving messages to and from the neurological system [74]. These blood cells act as robust systemic biomarkers of neurodegenerative diseases, reflecting the pathological characteristics of neural cells [75,76]. The best outcome proved that BDNF, produced in megakaryocytes and accumulated in platelets, is involved in the pathophysiology of depression by modulating thrombosis [71,77]. In addition, the neuronal protein reelin is found in blood platelets and plasma, where it controls arterial thrombosis, synaptic plasticity, and cell migration [78,79]. Essential for neuronal placement and laminar structure, reelin is a secreted ligand produced in the discrete layers of the developing brain [80].

Glial cells are indispensable for ideal neuronal growth, synaptic function, and formation within the CNS. Astrocytes release various substances that control the connectivity between neurons and circuit development [81]. BDNF is a miscible polypeptide secreted and produced by neurally connected effector tissues, glial cells, and neurons [82]. Furthermore, glial cells and neurons use BDNF as a key modulator of signaling [83]. Glial cells are the primary origin of BDNF production and secretion. Consequently, glial cell-derived BDNF also boosts excitatory neuron NMDA receptors, which increases excitatory neuron synaptic activity and decreases inhibitory neuron function by transiently depolarizing the reversal potential (EGABA), and reducing presynaptic GABAAR (GGABA) conductance and inhibitory neuron K-Cl co-transporter (KCC2) action in the spinal dorsal horn, which causes NP [84]. The CNS function is closely linked to BDNF expression. BDNF is primarily synthesized and expressed in various neuronal cells of the brain, including sensory and motor neurons [85]. As neurodegeneration may be largely influenced by neuroinflammation, a mounting body of evidence suggests that GFL receptors are present in both glial and peripheral immune cells. Consequently, by directing interventions towards GDNF family ligands (GFL) receptors using either proteins or small molecules, it is plausible to directly inhibit the activation of these cells, thereby potentially diminishing neuroinflammation [86]. Glial cells and neurons express a large amount of BDNF in the spinal cord and brain [87]. Glial cells were thought to merely provide structural and functional support to neurons, which were considered to perform the most significant activities [88]. Glial cells can protect and support neurons, but they also play a vital role in the pathophysiology of neurodegenerative disorders due to their dysregulation and abnormal activation. Researchers have developed a method to deliver bone marrow stem cells that undergo a genetic change to carry BDNF into the central nervous system. Additionally, they investigated how BDNF therapy alters outcomes for experimental autoimmune encephalomyelitis (EAE), an animal model of MS [89].

## 4. Secretion Mechanisms of BDNF

BDNF is produced and folded in the endoplasmic reticulum (ER) from its glycosylated precursor, pre-proBDNF. This protein is highly conserved and is primarily responsible for the biological effects of BDNF. It consists of a mature domain, pro-domain, and short signal peptide. Subsequently, proBDNF is transferred to the Golgi apparatus [90], and neuropeptide targeting begins with the synthesis of proproteins in the ER, from where they are transported to the Golgi apparatus via transport vesicles and enclosed in large granular vesicles (LGVs). Within the cell bodies, LGVs that contain only a single component of the cocktail are considered immature, as they are likely to be imposed on terminals after integrating with other peptide(s) [91]. These proproteins serve as precursors of mature proteins and may have their own biological functions. However, they typically undergo multiple post-translational processing steps to produce biologically active peptides [92]. The Golgi apparatus has multiple functions. It also serves as a hub for intracellular membrane trafficking, and is essential for processing and arranging recycled and newly generated proteins and lipids as they move toward their intended destinations. In general, the Golgi complex is involved in cellular function, such as cell division and apoptosis, and in the development and preservation of cell compartmentalization [93]. The hypothalamic–neurohypophysial system’s peptidergic neurons are the most well-studied of the various kinds of peptidergic neurons found in the CNS and PNS from the morphological, functional, and biochemical perspectives. Neurosecretory neurons located in the paraventricular and supraoptic nuclei of the hypothalamus generate the peptide hormones vasopressin and oxytocin, and the neurophysins that are linked to these hormones [94]. Temporal characteristics of hippocampal neuronal activity are encoded by BDNF release. The necessity of calcium mobilization from intracellular reserves and calcium influx through N-type calcium channels unambiguously shows how calcium-induced calcium release functions in the activity-dependent synthesis of BDNF [95].

DCVs also localize to synapses. They are produced in the cell body and sent to synapses, in contrast to synaptic vesicles, and contain neuropeptides and peptidergic neurotransmitters [96]. DCVs are responsible for the storage and secretion of biogenic amines, peptides, and neurotrophins. These include insulin secreted from DCVs in pancreatic β cells and catecholamines produced from adrenal chromaffin cells. DCVs have been extensively studied in several tissues [97]. Each neurotrophin family member and BDNF co-localize in the ER and Golgi apparatus of cell bodies. They are also dispersed throughout the large DCVs found in the axons and dendrites of neurons. This demonstrates how shared vesicles can release BDNF, which is induced by depolarization through a controlled secretory pathway [98]. Lysosomes are the primary source of vesicular ATP secretion [99]. Nevertheless, astrocyte-released ATP is crucial for maintaining CNS homeostasis because it controls the transmission of Ca^2+^ waves, OS, and synaptic activity in neurons, which affect neuronal plasticity [100]. Of all the compounds identified in DCVs, ATP has garnered the greatest attention due to its strong transmitter properties that impact glial and neuronal signaling, and pancreatic β cell behavior [101]. Interestingly, several cells exhibit GTP-dependent DCV secretion, indicating that vesicle release can be triggered by signals other than Ca^2+^. Non-hydrolyzable GTP initiates secretion in mast, chromaffin, and pancreatic β cells without requiring Ca^2+^ [97]. Thus, DCVs play an important role as carriers for the storage and regulated release of BDNF from the neurons.

## 5. Cellular Stimulation of BDNF Secretion

Different cellular stimuli control the release of BDNF. The following are some of the main cellular pathways leading to the release of BDNF:

### 5.1. Calcium Influx

Calcium is one of the main factors that regulate BDNF signaling pathways. In pre- and postsynaptic neurons, calcium triggers the activity-dependent release and synthesis of BDNF, and aids the promotion of appropriate BDNF TrkBr signaling [102]. The functionality of BDNF relies significantly on appropriate calcium signaling [103]. N-methyl-d-aspartate receptors (NMDARs) are among the key elements contributing to calcium-BDNF signaling. Activity-dependent BDNF expression requires NMDARs [104]. The binding of BDNF to TrkB amplifies the activation of NMDARs after mature BDNF is released into the synaptic cleft [105]. Moreover, BDNF is retained in a population of DCV, distinct from SP, that are exocytosed after burst stimulation. It is possible that the high-frequency action potentials of every burst cause a significant increase in calcium concentrations in the presynaptic terminals, and that BDNF release depends on these higher calcium levels [106]. NMDAR modulators stimulate BDNF expression and exert rapid antidepressant effects, suggesting that NMDAR dysregulation leads to depression [107]. The stimulation of NMDARs by BDNF amplifies the intracellular influx of calcium, thereby promoting the synthesis of more BDNF [108]. The intricate relationship between these molecules in neural transmission is highlighted by the interaction between BDNF and NMDARs, which strengthens the signaling cascade. Overall, the significance of these pathways in synaptic transmission and neural plasticity is highlighted by the interactions among BDNF, NMDARs, and calcium signaling.

### 5.2. Cyclic Adenosine Monophosphate (cAMP) Signaling

Transmembrane or soluble adenylate cyclase (AC) converts ATP to cAMP, which is a signaling molecule. Ion channels, exchange proteins activated by cAMP (EPAC), and cAMP-dependent protein kinases (PKA) are examples of the downstream effectors of cAMP [109]. RNA silencing revealed that PKA inhibition had no effect on BDNF secretion, whereas Epac2, but not Epac1, is crucial for hyperoxia-induced BDNF secretion. Consequently, TrkB and BDNF siRNAs blocked the autocrine action of BDNF, which increased the cAMP levels in airway smooth muscle (ASM). cAMP-mediated Epac2 activation in ASM affects BDNF release. This creates a positive feedback loop, whereby BDNF elevation increases cAMP levels. This mechanism likely increases ASM contractility and proliferation by maintaining BDNF production following hypertrophic stimulation [110]. The expression of genes essential for dopaminergic neurons is regulated by an intracellular protein known as cAMP response element binding protein (CREB) [111]. These findings suggest that BDNF’s control over dendritic development necessitates both the fostering of CREB phosphorylation by BDNF and the induction of CRTC1 nuclear translocation by glutamate through NMDAR activation [112]. cAMP, which is regulated by G protein-coupled receptors (GPCRs), plays a distinctive role in the CNS by overseeing neuronal growth and development, synaptic plasticity, neurogenesis, and memory consolidation. It primarily enhances extracellular signals through PKA activation, initiating a cascade of biochemical and physiological responses [113]. Furthermore, CREB, a key substrate of PKA, can be phosphorylated. CREB phosphorylation controls hippocampal synaptic plasticity by altering the transcription of target genes [114]. In animal models of depression, PKA triggered target gene transcription in the nucleus by activating CREB, which leads to the regulation of excitement, development, apoptosis, and synaptic plasticity of hippocampal neurons [115]. However, the cAMP signaling system plays a crucial role in regulating the release of BDNF. Increased intracellular cAMP levels result from the activation of GPCRs, and PKA is subsequently activated. The phosphorylation of PKA proteins leads to the production of BDNF, ultimately influencing synaptic transmission and plasticity. This signaling pathway highlights the intricate mechanisms by which cellular signaling affects neuronal function and connectivity.

### 5.3. Glutamatergic Signaling

The most significant excitatory neurotransmitter in the CNS, glutamate, is necessary for excitatory synaptic transmission because it governs glutamate signaling. Dysfunction of the glutamate pathway plays a role in the development of neuropsychiatric and neurodegenerative disorders [116]. Ca^2+^ release from internal Ca^2+^ stores is necessary for glutamate-induced BDNF release from brain slices or primary neuronal cultures, and extracellular Ca^2+^ is necessary for BDNF secretion in response to action potential [117]. Based on sequence homology and G-protein coupling, the metabotropic glutamate receptor (mGluR) family is divided into the following three groups: Group I consisting of mGluR1 and 5; Group II consisting of mGluR2 and 3; and Group III consisting of mGluRs 4, 6, 7, and 8 [118]. Whereas, the mGluR1-dependent activation of PLC induces BDNF release in hippocampal neurons [119]. The expression of target genes involved in dendritic formation is regulated by CREB through a cooperative interplay between BDNF- and glutamate-mediated signaling. BDNF controls the expression and phosphorylation of NMDA subunits. Synaptic transmission and plasticity modulation mechanisms in the CNS are significantly affected by the coordinated actions of glutamate and BDNF. Specifically, glutamate and BDNF co-regulate each other in a way that promotes glutamate release and, in turn, increases BDNF transcription and secretion [112].

### 5.4. Depolarization

Synaptotagmin (Syt) is the protein responsible for interacting with soluble N-ethylmaleimide-sensitive-factor attachment protein receptor (SNARE) proteins. Synaptotagmins are typically localized within the vesicular membrane. Among this protein family is synaptotagmin-4 (Syt-4), which shows increased expression in response to activity and seizures, and is localized to BDNF vesicles. Syt-4 plays a role in modulating BDNF release by inhibiting the fusion step, thereby reducing depolarization-induced BDNF release [120]. The production of BDNF by neurons is dependent on either prolonged depolarization or persistent firing of action potentials [121]. Different cell types express and release BDNF, and various stimuli can trigger its regulated release. Patterns of neuronal electrical activity, such as prolonged depolarization, high-frequency stimulation (HFS), and theta-burst stimulation (TBS), are the most extensively studied stimuli known to induce BDNF release in developing and mature neurons [122]. Increased expression of BDNF has been noted in various classes of antidepressants, including 5-HT and norepinephrine selective reuptake inhibitors, tricyclic antidepressants, monoamine oxidase inhibitors, and electroconvulsive seizures (ECS). Notably, ECS administration, which rapidly induces neuronal depolarization, leads to an immediate increase in BDNF expression in both the hippocampus and prefrontal cortex (PFC) [123]. The level of neural networks determines the amount of BDNF secretion, because immature and mature synaptic circuits require different electrical activity patterns to trigger BDNF release. Three distinct patterns of electrical activity relevant to the formation of synaptic circuits in the growing hippocampus and cortical networks have been identified: giant depolarization potentials (GDPs), synchronized plateau assemblies, and short L-type voltage-gated calcium channels (VGCCs)-mediated spikes [124]. Various patterns of electrical activity rely on the activation of VGCCs, and the release of BDNF depends on the entry of Ca^2+^ through these channels [125]. Thus, depolarization aids BDNF release by promoting the activation of VGCCs, which allows calcium ions to enter the cell. This influx of calcium triggers the release of BDNF because its secretion is dependent on calcium signaling.

## 6. Neuroprotective Effects of Phytochemicals as Potential Medicines

Cognitive impairment is a substantial health issue in the 21st century. Parkinsonism and other neuropsychiatric and neurodegenerative illnesses, including depression, schizophrenia, dementia due to AD, cerebrovascular impairment, seizure disorders, head injuries, and seizures can have a severely debilitating effect on one’s ability to function. The prevalence of neurological illnesses is sharply increasing because of their complicated pathophysiology and the lack of disease-modifying treatments. Numerous efforts have been made to discover novel compounds that can treat neurological disorders. The potential neuroprotective benefits of phytochemicals and natural compounds present in plants have garnered increasing attention. Among phytochemicals, indole-3-carbinol (I3C) is particularly notable for its capacity to promote neuroprotection; it has anti-inflammatory and antioxidant properties in addition to influencing the brain’s neurotransmitter systems. Natural sources of I3C include cruciferous vegetables like kale, broccoli, cabbage, and Brussels sprouts. However, I3C levels in these foods are rather low. To obtain therapeutic levels of I3C, one would have to consume considerable amounts of these vegetables, which is not always possible. Supplements give a concentrated and consistent dose of I3C, ensuring that an individual receives enough to potentially benefit from its neuroprotective properties. However, these distinctions are harder to discern in the later stages of dementia [126]. According to neuropathological studies, cortical Lewy body/neuritic disease is more prevalent and severe in PDD than in PD without dementia [127]. Furthermore, cholinergic deficiencies occur in PDD, with higher levels in patients with a longer duration of parkinsonism prior to dementia and a lower cortical and limbic Lewy body/neuritic burden, and are attributed to neuronal death in basal forebrain cholinergic nuclei. The occurrence of a cortical cholinergic deficiency in PDD patients suggests that cholinesterase inhibitor therapy may be advantageous [128].

Studies and research are currently underway, and the expectation that I3C or its metabolites and derivatives will one day serve as the foundation for the development of novel medications with great efficacy and selectivity while causing minimal or no adverse effects is becoming more concrete. The 3,3′-DIMs, in particular, have been the subject of extensive research. While only a few 2,3′-DIM compounds have been examined and identified as an AhR receptor agonism, they hold great promise for future research and applications [129].

In this comprehensive review article, the neuroprotective efficacy of I3C was explored, emphasizing its role in mitigating OS and inflammation, and in regulating neurotransmitter systems in the brain.

### 6.1. I3C

**Figure 2:**
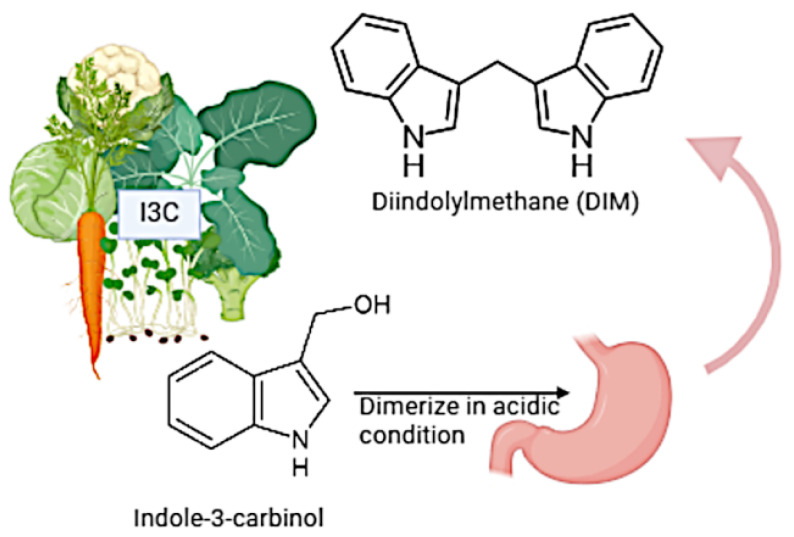
Different sources of I3C and its dimerization product, such as DIM, under acidic conditions (created by biorender).

I3C (C9H9NO) is a phytochemical naturally present in cruciferous vegetables of the Brassicaceae family and is formed by the degradation of glucobrassicin, a specific glucosinolate compound present in raw vegetables (Figure 2). Cruciferous vegetables, such as broccoli, cauliflower, cabbage, and Brussels sprouts, contain heterocyclic and bioactive compounds, I3C and 3,3′-diindolylmethane (DIM).

**Figure 3:**
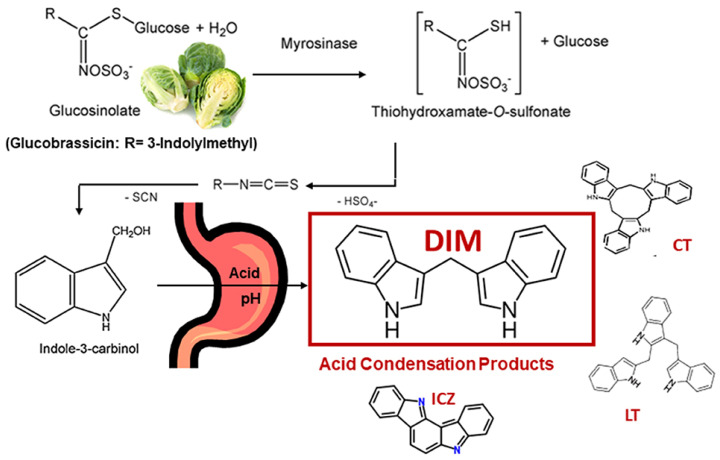
The cleavage of glucobrassicin with the action of Myrosinase reveals the formation of I3C and acid condensation products, including DIM. Reprinted from [132] Copyright [2021/Frontiers] [Frontiers in Nutrition].

The hydrolysis products of glucosinolates, such as isothiocyanates and indoles, are abundant in these vegetables [130] (Figure 3). A specific type of β-thioglucosidases activates glucosinolate, a sulfur-rich compound with various metabolic and biological properties. When chewing or chopping damages the plant cells, glucosinolate is produced, and myrosinase comes into contact with glucosinolate and catalyzes its hydrolysis [131].

**Figure 4:**
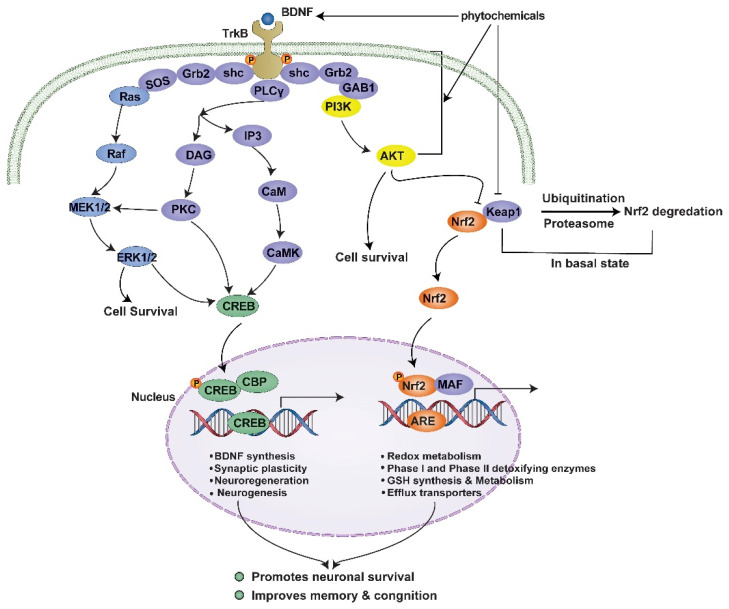
Phytochemical-mediated signaling pathways are triggered by the interaction between BDNF and TrkB, resulting in cellular reactions that safeguard neurons and improve cognitive abilities. Reprinted from [4]. Copyright [2020/Frontiers] [Frontiers In Molecular Neuroscience/Frontiers].

The health effects of glucosinolate hydrolysis products, such as isothiocyanates and I3C, are widely recognized, and these compounds may even mitigate neurodegenerative diseases [133]. DIM may shield neural cells of the brain tissue from ischemia and inflammation; DIM activated the TrkB/Akt signal pathway in hippocampus neuronal cells exposed to OS, resulting in the production of BDNF and antioxidant enzymes, and a neuroprotective effect along with the ability to preserve the cholinergic system in mice exposed to scopolamine [21]. The in vitro results indicated that I3C significantly increased the levels of the antioxidant-associated genes NAD(P)H quinone dehydrogenase 1 (NQO1), heme oxygenase 1 (HMOX1), and cationic amino acid transporter 1 (CAT1) in BV-2 cells, and potently reduced the production of lipopolysaccharide (LPS)-induced nitric oxide synthase (I-NOS), IL-1ß, NLRP3, IL-6, and chemokine (C-C motif) ligand 2 (CCL2) by proinflammatory genes. In addition, I3C decreased migration, phagocytosis, and NO secretion caused by LPS. Figure 4 represents the intricate interplay of phytochemical-mediated signaling pathways that are triggered by the interaction between BDNF and TrkB, resulting in cellular reactions that safeguard neurons and improve cognitive abilities.

Based on these findings, research hypothesized that DIM may protect hippocampal neuronal cells from oxidative stress-induced apoptosis by keeping both the TrkB/CREB/BDNF and the Akt/Nrf2/ARE pathways active. DIM protected hippocampus neurons from oxidative stress-induced death by increasing the expression of both BDNF and antioxidant enzymes like HO-1, NQO-1, and GCLC. In the signaling cascade, TrkB activation by BDNF causes Akt phosphorylation, and then activated Akt can create BDNF in neural cells by activating CREB [134].

Previously identified gene-regulatory events were substantially inhibited by aryl hydrocarbon receptor (AhR) knockdown using siRNA. In vivo experiments showed that I3C treatment decreased light damage-induced I-NOS, IL-1ß, NLRP3, IL-6, and CCL2 transcripts, and CCL2, I-NOS, IL-1ß, and p-NFkBp65 protein levels in mice [135]. I3C has promising potential for reducing the inflammatory, oxidative, and proarrhythmic processes associated with hypertension. The key mechanisms underlying its protective effects include the modulation of NO levels and upregulation of heat shock protein 70 [136].

### 6.2. Application of Naturally Derived I3C and Its Derivatives in Treating Depression

In depression pathogenesis, elevated levels of proinflammatory cytokines in the brain are recognized for their role in impairing neuronal function by reducing the expression of BDNF [137]. Studies have shown that I3C counteracted the BDNF levels in the PFC and hippocampal regions, which were decreased by chronic social defeat stress (CSDS). This suggests that a possible cause for the increased BDNF levels observed after repeated I3C treatment is a decrease in proinflammatory cytokines [138]. Increased levels of proinflammatory cytokines can also induce a chain reaction known as oxido-nitrosative stress, whereby elevated nitrite levels and ROS probably promote depression by inhibiting BDNF expression [139]. Reducing the advancement of neuroinflammation may coincide with increased BDNF expression [140]. This suggests that, in addition to counteracting the overactive neuroinflammatory response, protection against oxide-nitrosative stress may be a possible mechanism for I3C’s antidepressant-like effects. DIM, a dimer of I3C, demonstrated a dose-dependent increase in the expression of phosphorylated TrkB, CREB, Akt, and BDNF, along with the upregulation of antioxidant enzymes, such as glutamate-cysteine ligase catalytic subunit (GCLC), NAD(P)H quinone oxidoreductase 1 (NQO-1) in glutamate-treated HT-22 cells, and HO-1. Moreover, DIM facilitated the nuclear translocation of Nrf2 in HT-22 cells treated with glutamate.

Notably, at a concentration of 40 µM, DIM nearly completely reinstated the expression of all proteins. These findings suggest that DIM has the potential to stimulate the Akt/Nrf2/ARE and TrkB/CREB/BDNF pathways in HT-22 cells exposed to oxidative stress. Hence, the neuroprotective effects of DIM may be closely linked to the activation of these signaling pathways [21].

### 6.3. I3C and Its Role in Neurological Diseases

Recently, phytochemicals have attracted considerable attention owing to their potential to prevent neurodegenerative disorders. Although there is still much to learn regarding the role of phytochemicals in neurodegenerative diseases, a growing body of preclinical and observational research indicates that a high-phytochemical diet may have neuroprotective effects and lower the risk of contracting these crippling illnesses. Both I3C and DIM decrease LPS-stimulated inflammatory responses in murine macrophages, indicating that they could additionally prevent neuroinflammation-mediated neurodegeneration and microglial hyperactivation [141]. DIM, a byproduct of I3C, prevents oxidative stress-induced death in neuronal cells by controlling the expression of proteins linked to apoptosis in glutamate-treated HT-22 cells. It can safeguard against OS-induced neurodegeneration by triggering the production of BDNF and antioxidant enzymes while strengthening the stimulation of the TrkB cascade [21].

Continuous degradation of the structure and function of the nervous system is a hallmark of neurodegenerative disorders, including AD, PD, and HD. Phytochemicals have drawn increasing attention in recent years due to their bioactive qualities as potential medicinal agents. The following table (Table 1) provides an extensive overview of I3C and its derivatives, along with information on how each specifically operates in relation to neurodegenerative disorders. These properties include neuroprotective, anti-inflammatory, and antioxidant properties. Phytochemicals affect several molecular pathways essential for the pathogenesis of neurodegenerative illnesses. Understanding the molecular pathways by which phytochemicals function is essential for designing targeted therapeutics. These processes include increased levels of neurotrophic factors, suppression of OS, control of inflammatory responses, and the avoidance of protein aggregation. By clarifying these pathways, researchers can gain a deeper understanding of how phytochemicals support neuroprotection and may even impede the progression of illness.

#### 6.3.1. I3C and PD

PD is the second most common progressive neurological disease worldwide. Despite extensive research, there is no effective treatment that can halt disease progression. Motor dysfunction, including bradykinesia, stiffness, postural instability, and resting tremors, is a clinical hallmark of PD [147]. A previous study explored the protective effects of I3C in a rat model of chronic rotenone (ROT)-induced Parkinson’s disease. Treatment with ROT alone caused substantial weight loss and severe motor impairment, including rigidity, reduced movement, and balance problems. Among the doses tested, the highest dose of I3C (100 mg/kg) was most effective in preventing ROT-induced motor deficits. It also mitigated depletions in striatal dopamine levels, weight loss, neurodegeneration, decreases in tyrosine hydroxylase (TH) expression, and increases in α-Syn expression in both the midbrain and striatum [148]. Furthermore, in a rat model of clonidine-induced depression, I3C demonstrated a strong neuroprotective effect [149]. I3C is also protected against LPS-induced neuroinflammation in the PD model [143].

#### 6.3.2. I3C and AD

AD is a complex neurological condition associated with various factors. Alterations in the gut microbiota composition and microbial metabolites may contribute to the development of several neurological disorders, including AD [150]. It is defined by the presence of external senile plaques and intracellular neurofibrillary tangles, which consist of amyloid-β and hyperphosphorylated tau proteins [151]. I3C derivatives serve as agonists of AhR, a vital environmental polyaromatic chemical sensor that influences AhR target gene expression and intestinal microbiota composition [152]. I3C acts as an AhR agonist and has numerous biological activities [153]. AD, characterized by Aβ plaques and neurofibrillary tangles, is the most prevalent type of neurodegenerative disease. Remarkably, neprilysin, a key endogenous Aβ catabolic enzyme, exhibited markedly elevated expression and activity in response to I3C [144]. I3C has garnered attention for its potential role in AD via its interaction with AhR. Host cells of the gastrointestinal tract and bacteria interact closely to promote serotonin production by enterochromaffin cells. Tryptophan can be converted into tryptamine by certain microorganisms, which triggers AhR signaling. Interactions between the gut microbiota and host, such as inflammation, trigger the indoleamine 2,3-dioxygenase 1/tryptophan dioxygenase-induced (IDO1/TDO-induced) kynurenine (KYN) pathway, which produces new AhR agonists. IDO1 expression is then stimulated via AhR signaling, thereby creating a positive feedback loop [154]. AhR activation modulates immune responses in the brain and regulates the production of inflammatory mediators implicated in AD pathology.

#### 6.3.3. I3C and ALSs

ALS, sometimes called Lou Gehrig’s disease or motor neuron disease, is a degenerative condition of the spinal cord and nerve tissues that causes paralysis and muscular weakness [155]. In ALS, motor neurons degrade gradually before they die. When motor neurons suffer damage or death, the signals that should be sent to the brain are no longer delivered [156]. Recent preclinical and clinical studies have identified compounds in cruciferous vegetables that may serve as neuroprotective agents. Cruciferous vegetables, such as collard greens, cabbage, turnips, and Brussels sprouts, are rich in nutrients and phytochemicals, such as I3C/DIM, which contain sulforaphane (SFN), and have the ability to reduce mutations in TAR DNA-binding protein-43, a factor associated with motor neuron dysfunction in ALS. This mechanism is thought to be mediated by activation of the NRF2/ARE pathway [157]. In addition, treatment with SFN protects the neurons from various neurotoxins, including dopamine, glutamate, arsenic, okadaic acid, tributyltin, and LPS [158]. In summary, the identification of neuroprotective qualities of compounds in cruciferous vegetables highlights the significance of further research into dietary approaches for treating this condition and is a promising strategy for the development of therapeutic techniques against ALS.

#### 6.3.4. I3C and HD

Huntington’s disease (HD) is a dominant mutant neurodegenerative disorder that causes mobility disorders, psychological signs, and cognitive impairment. The HD monogenic mutation encodes a Huntingtin (HTT) protein variation. The condition is caused by a CAG repeat extension that results in an excessively long polyglutamine (Q) stretch at the N-terminus of HTT, possibly conferring a deleterious gain of function on the mutant polypeptide. There are presently no viable disease-modifying or preventative treatments for HD [159].

Most importantly, small-molecule inhibitors, such as I3C, can inhibit the enzymatic activity of WW domain-containing E3 ubiquitin protein ligase 1 (WWP1), which is responsible for causing several neurological disorders. WWP1 is a member of the homologous E6-associated protein carboxyl terminus (HECT) E3 ligase family and has been implicated in several human diseases, including cancer, age-associated osteogenic disorders, neurodevelopmental disorders, and infectious diseases [160]. Caballero et al. demonstrated that WWP1 can induce ubiquitination of the T-type channel Cav3.2. This process subsequently disrupts ubiquitin-specific peptidase 5 (USP5) function and contributes to neuropathic and inflammatory pain [161]. Research suggests that WWP1 plays a role in the initiation of HD by modifying aggregation, increasing the expression of mutant huntingtin protein (mHtt), and inducing cell toxicity via polyubiquitination of mHtt through K63 [162].

#### 6.3.5. I3C and Multiple Sclerosis (MS)

MS is an incurable autoimmune disease that occurs when the immune system identifies the myelin sheath that surrounds the neurons as another foreign substance, causing an unintentional chain reaction of pathogenic inflammation in the CNS. In progressive MS, inflammatory cascades create plaques and lesions in the CNS composed of demyelinated tissue that impair sensory tissue function [163]. Decreased levels of circulating AhR in individuals with MS compared to healthy controls imply a potential role for AhR in the pathogenesis of MS [164]. In an experimental autoimmune encephalitis (EAE) model of MS [165], knockdown of AhR exacerbated disease severity, whereas activation of AhR using agonists, such as 2,3,7,8-tetrachlorodibenzo-p-dioxin (TCDD), I3C, and DIM suppressed EAE progression. This suppression was achieved by enhancing forkhead box P3 (FOXP3) expression, promoting the expansion of anti-inflammatory regulatory T cells (Treg), and reducing the expansion of proinflammatory Th17 cells [164,166,167]. Hence, targeting AhR with compounds, such as I3C and DIM, could be a promising therapeutic approach for the development of drugs for MS.

## 7. Preclinical Studies on I3C and Neuroprotection

A preclinical study revealed that I3C reduced serum S100B levels in rats with cerebral ischemia-reperfusion injury (CIRI), indicating its potential anti-inflammatory effects. Additionally, administration of 50 mg/kg and 25 mg/kg I3C correlated with decreased Il1b, Il6, and Nfkb2 gene transcripts, reduced myeloperoxidase (MPO) activity, and lowered expression of the p65 subunit in brain tissue. These findings suggest that I3C exerts neuroprotective effects by alleviating OS and suppressing inflammation in CIRI. Furthermore, histopathological analysis confirmed the neuroprotective effects of I3C on cortical and hippocampal tissue morphology [168].

Studies have explored the effects of I3C on neonatal anoxia-induced brain injury and subsequent neurodevelopmental deficits. In this study, rat pups were exposed to two episodes of anoxia, each lasting 10 min, with a 24-h interval between episodes. Anoxia was induced by replacing air with 100% nitrogen. I3C administration was started within 30 min after the second anoxic episode, starting on a postnatal day 3 (PND 3) and was continued until PND 9. Treatment with I3C resulted in a dose-dependent improvement in neurodevelopmental deficits and somatic growth in anoxic pups. Furthermore, I3C treatment was associated with enhanced mitochondrial function, as evidenced by improvements in mitochondrial membrane potential (MMP), mitochondrial electron transport chain (ETC) enzymes, and antioxidant levels. Additionally, I3C inhibited the opening of the mitochondrial permeability transition pore (MPTP) and the release of cytochrome C in anoxic pups. Moreover, I3C administration reduced elevated cortical levels of hypoxia-inducible factor 1-alpha (HIF-1α) in neonatal anoxic pups [169].

The study suggests I3C protected against clonidine-induced depression-like behaviors in mice. Additionally, the neuroprotective mechanisms underlying this effect, including antioxidant, anti-inflammatory, and modulatory effects on monoamine levels in brain tissue were examined. I3C was administered orally at a dose of 50 mg/kg daily for two weeks, starting seven days before clonidine administration. Clonidine significantly induced behavioral changes, OS, inflammation, apoptosis, and decreased monoamine levels. However, I3C pretreatment effectively attenuated the effects induced by clonidine, suggesting its potential therapeutic value in depression. Additionally, brain specimens from rats treated with I3C alone showed mild diffuse gliosis [169].

Administering I3C to sham and middle cerebral artery occlusion (MCAO) rats did not cause any changes in pharmacokinetic parameters, including tissue distribution, clearance, bioavailability, volume of distribution, area under the curve, mean residence time, maximum plasma concentration (Cmax), or time to achieve Cmax. Oral administration of I3C led to higher levels of DIM in the plasma (five-fold), brain (four-fold), and cerebrospinal fluid (CSF) (two–three-fold) than intravenous administration. Furthermore, orally delivered I3C significantly mitigated neurological deficits, reduced brain infarction (by 20%), blood–brain barrier leakage (by 15 μg/g), and decreased brain water content (by 75%) in MCAO rats compared to intravenous administration [164].

## 8. Conclusions and Future Prospective

I3C, a bioactive substance present in cruciferous vegetables, can modify a number of biological processes that are essential for neuroprotection and general brain health. The ability of I3C to affect OS, inflammation and apoptosis, and to modulate brain-BDNF, is an important element in the pathophysiology of neurodegenerative diseases, and is largely responsible for its neuroprotective benefits. BDNF is an essential neurotrophin for the growth, survival, and operation of neurons. I3C increases BDNF expression, which promotes synaptic plasticity and neuronal survival, and the effects of I3C on the nervous system and cognition may be facilitated by this upregulation, thereby presenting treatment opportunities for diseases, such as PD, AD, and other neurodegenerative disorders. To maximize the therapeutic potential of I3C and enable novel treatments in neurology, further investigations into its safety, efficacy, and best delivery strategies are necessary. I3C plays various biological roles, demonstrating its potential as a flexible therapeutic agent for a broad spectrum of prevalent and atypical neurological disorders. In conclusion, I3C exhibits great potential as a neuroprotective drug and BDNF modulator, with applications ranging from cancer prevention and treatment to neuroprotection. To thoroughly comprehend its therapeutic potential and integrate these findings into useful medical applications, further investigation of its mechanisms of action and clinical efficacy is required.

Future possibilities for the treatment of neurological illnesses with I3C, DIM, and their derivatives are promising. These substances have potent antioxidant, anti-inflammatory, and neuroprotective properties, all of which are essential in the fight against neurodegenerative diseases. Clinical trials and further research may lead to the integration of DIM and I3C into tailored and efficient treatment plans for various neurological disorders. Understanding the intricate interplay between glial cells and neurons under these conditions is crucial for developing effective therapeutic approaches. According to Present Supplementary Guidance for I3C, for general health purposes common dosages fall between 200 and 400 mg daily, whereas for DIM normal dosages fall between 100 and 300 mg daily. The investigation into the application of DIM, I3C, and their derivatives to neurological disorders is still in its early stages. It is still unknown what dosages are appropriate for treating neurological conditions. Future research endeavors must ascertain the most effective and safest dosages, taking into account variables such as bioavailability, blood–brain barrier crossing capabilities, and possible drug interactions. Depending on the disease being treated, doses in therapeutic settings can differ greatly. Higher dosages, as opposed to general supplements for immune support or hormonal balance, might be investigated in cancer trials, for instance.

## Figures and Tables

**Table 1:** Indole-3-carbinol and its signaling protein-inducing derivatives are involved in neurological function.

Target Proteins	Regulatory Metabolites	Impact on Neurological Function	Active Concentration of I3C/DIM	References
T-regs Th17	Activation of AhR induces the generation of T-regs along with Th17 suppression.	Pre-administration of I3C or DIM in EAE mice resulted in the complete prevention of clinical symptoms and cellular infiltration into the CNS. Additionally, subsequent treatment with I3C or DIM following EAE onset demonstrated significant efficacy in lowering the overall severity of the disease. Represent innovative therapeutic options for attenuating neuroinflammation.	20 mg·kg^−1^ I3C or DIM	[142]
Inflammatory cytokines: TNF-α and IL-6	Prolonged administration of I3C over a 21-day period in rats treated with intranigral LPS resulted in notable enhancements in motor skills, coordination, and learning and memory abilities. These improvements correlated with a reduction in the levels of inflammatory cytokines, including TNF-α and IL-6.	The findings suggest that I3C shows promise as a therapeutic agent for delaying neurodegeneration in neurons affected by Parkinson’s disease, leading to enhancements in both motor and cognitive functions. I3C may hold promise as a therapeutic intervention for averting neurodegeneration in neurons associated with Parkinson’s disease, thereby enhancing both motor and cognitive functions.	50 mg/kg, chronic administration of I3C for 21 days	[143]
NEP	In N2a cells and APP/PS1 mice, activating AhR by the exogenous ligand indole-3-carbinol (I3C) or the endogenous ligands L-Kynurenine (L-KN) or FICZ dramatically boosts NEP expression and enzyme activity.	Controlling the neuronal expression of NEP I3C stimulated NEP activity, which in turn assisted with Aβ clearance. I3C activated AhR and elevated NEP, demonstrating a strong therapeutic effect on cognitive impairments. A novel approach to regulate NEP expression in neurons and that AhR could be a useful therapeutic target for Alzheimer’s disease treatment.	10 μM, I3C	[144]
P-glycoprotein	These substances may be substrates for P-glycoprotein and thus be susceptible to efflux transport at the blood–brain barrier.	Possessing the ability to alter brain activity, it can penetrate the blood–brain barrier and enter the brain through oral ingestion. The presence of I3C and its derivatives in this tissue raises the possibility that they have the ability to pass through the blood–brain barrier and influence the central nervous system pharmacologically.	250 mg/kg, I3C	[145]
Inhibited NF-κB	In vitro NDD/LPS-induced hyperactivation of microglia in BV-2 Microglia and mice (in vivo)	By reducing microglial hyperactivation and neuroinflammation, I3C/DIM could confer neuroprotective advantages and inhibit the development of neurogenerative disorders. Reduced apoptosis and neuroinflammation decreased hippocampal-activated microglial cells.	DIM (125 or 250 mg/kg), DIM (10, 20, and 40 μM), I3C (10, 20, and 40 Mm)	[146]
